# Scaling-up primary health care-based prevention and management of heavy drinking at the municipal level in middle-income countries in Latin America: Background and protocol for a three-country quasi-experimental study

**DOI:** 10.12688/f1000research.11173.3

**Published:** 2017-11-13

**Authors:** Peter Anderson, Amy O'Donnell, Eileen Kaner, Antoni Gual, Bernd Schulte, Augusto Pérez Gómez, Hein de Vries, Guillermina Natera Rey, Jürgen Rehm

**Affiliations:** 1 Institute of Health & Society, Newcastle University, Newcastle upon Tyne, NE2 4AX, UK; 2 Faculty of Health, Medicine and Life Sciences, Maastricht University, Maastricht, 6221 HA, Netherlands; 3 Addictions Unit, Psychiatry Dept, Hospital Clínic of Barcelona, Barcelona, 08036, Spain; 4 Institut d’Investigacions Biomèdiques August Pi Sunyer (IDIBAPS), Barcelona, 08036, Spain; 5 Red de Trastornos Adictivo, Instituto de Salud Carlos III, Madrid, 28029, Spain; 6 Centre for Interdisciplinary Addiction Research, University Medical Center Hamburg-Eppendorf, Hamburg, 20246, Germany; 7 Corporación Nuevos Rumbos, Bogotá, Colombia; 8 Department of Health Promotion, Maastricht University, Maastricht, 6200, Netherlands; 9 Instituto Nacional de Psiquiatría Ramón de la Fuente Muñiz, Mexico City, 14370, Mexico; 10 Institute for Mental Health Policy Research, Toronto, ON, M5S 2S1, Canada; 11 Dalla Lana School of Public Health, University of Toronto, Toronto, ON, M5T 3M7, Canada; 12 Department of Psychiatry, University of Toronto, Toronto, ON, M5T 3M7, Canada; 13 Institute for Clinical Psychology and Psychotherapy, TU Dresden, Dresden, 01187, Germany

**Keywords:** Scale-up, implementation, primary health care, cities, alcohol use disorder, harmful use of alcohol, heavy drinking, training and support

## Abstract

**Background**: While primary health care (PHC)-based prevention and management of heavy drinking is clinically effective and cost-effective, it remains poorly implemented in routine practice. Systematic reviews and multi-country studies have demonstrated the ability of training and support programmes to increase PHC-based screening and brief advice activity to reduce heavy drinking. However, gains have been only modest and short term at best. WHO studies have concluded that a more effective uptake could be achieved by embedding PHC activity within broader community and municipal support.

**Protocol**: A quasi-experimental study will compare PHC-based prevention and management of heavy drinking in three intervention cities from Colombia, Mexico and Peru with three comparator cities from the same countries. In the implementation cities, primary health care units (PHCUs) will receive training embedded within ongoing supportive municipal action over an 18-month implementation period. In the comparator cities, practice as usual will continue at both municipal and PHCU levels. The primary outcome will be the proportion of consulting adult patients intervened with (screened and advice given to screen positives). The study is powered to detect a doubling of the outcome measure from an estimated 2.5/1,000 patients at baseline. Formal evaluation points will be at baseline, mid-point and end-point of the 18-month implementation period. We will present the ratio (plus 95% confidence interval) of the proportion of patients receiving intervention in the implementation cities with the proportions in the comparator cities. Full process evaluation will be undertaken, coupled with an analysis of potential contextual, financial and political-economy influencing factors.

**Discussion**: This multi-country study will test the extent to which embedding PHC-based prevention and management of alcohol use disorder with supportive municipal action leads to improved scale-up of more patients with heavy drinking receiving appropriate advice and treatment.

**Study status**: The four-year study will start on 1 ^st^ December 2017.

## Introduction

### Harm done by alcohol

Alcohol is a cause of a wide range of diseases and injuries, exacerbated by occasions of heavy drinking ^1^, resulting in it ranking as the ninth leading global risk-factor in 2015 for morbidity and premature death ^2^. Ranking increases to fourth in Colombia and Peru, and fifth in Mexico ^2^, the three Latin American countries addressed in this protocol.

The clinical condition of alcohol use disorder (AUD) ^3– 5^, which includes the harmful use of alcohol, is associated with considerable disability, morbidity, and mortality ^6, 7^. Worldwide in 2015, there were 63.5 million cases of AUD ^8^ (due to more restrictive definitions, this is lower than other estimates of 95 million cases ^9^), responsible for 137,500 deaths ^10^, 6.3 million years lived with disability ^8^, and 112 million disability adjusted life years ^11^.

### Sustainable development goals

Adverse impacts from AUD and the harmful use of alcohol are aggravated by lower socio-economic status ^12^. Impacts also extend beyond the individual drinker, with considerable costs borne by families, communities, health systems, and the wider economy ^7^. A large proportion of these costs are avertable ^13^. Tackling the multiple individual and societal level harms caused by AUD and the harmful use of alcohol is a global economic and public health priority, and essential for achieving global targets of reducing deaths from non-communicable diseases by 25% between 2010 and 2025 ^14^, more so as risk of exposure to harmful use of alcohol increases with increasing socio-economic status in low and middle income countries ^2^. Further, building on the global NCD framework ^15^ and the WHO global strategy ^16^, UN Sustainable Development Goals Target 3.5 is to strengthen the prevention and treatment of substance abuse, including narcotic drug abuse and harmful use of alcohol, with two proposed indicators: coverage of treatment interventions (pharmacological, psychosocial and rehabilitation and aftercare services) for substance use disorders (including AUD); and, per capita alcohol consumption ^17^.

### Heavy drinking

This protocol focuses on the prevention and management of heavy drinking, an understandable term to use when identifying at risk patients in primary health care (PHC) ^18– 21^. We base our definition of heavy drinking on the European Medicines Agency’s ‘threshold 1’, more than 60g of alcohol consumed on average a day by a man and more than 40g a day by a woman ^22^. These are the same levels as original descriptions used in global burden of disease studies ^23^. For practical purposes, we take the mid-point (50g a day) as our definition of heavy drinking. At this level of consumption, there is little difference in absolute risk (about 3.5%) of dying prematurely due to alcohol before the age of 70 years between men and women ^24^.

### Advice and treatment gap

Despite the fact that heavy drinking is one of the most important modifiable causes of premature morbidity and mortality ^25^, worldwide, although the data should be interpreted with caution, it is estimated by WHO that as many as four out of five heavy drinking individuals fail to receive the offer of appropriate advice or treatment ^26, 27^. In Mexico, the gap is nine out of ten ^28, 29^. The problem is not one of lack of effective treatment and prevention options ^30, 31^. A robust and extensive body of literature demonstrates the range of evidence-based strategies available to policy makers and practitioners seeking to reduce heavy drinking ^31, 32^. Questionnaire-based screening and brief advice programmes delivered in PHC are effective ^33^ and cost-effective ^34^ in reducing heavy drinking, even though the extent to which this evidence-base is grounded in efficacy (ideal world) or effectiveness (real world) trials is still debated in some academic circles ^35^. In addition to brief advice, treatment for AUD and harmful alcohol use include cognitive behavioural therapy and pharmacotherapy, both of which are found to be effective in reducing heavy drinking ^36– 39^. However, to date at least, these have failed to achieve widespread up-take ^31, 40, 41^.

The Organisation for Economic Co-operation and Development (OECD) has estimated that if the proportion of eligible patients receiving advice and treatment for heavy drinking increased to 30% of eligible patients, the prevalence of harmful use of alcohol could decrease by as much as 10–15% across OECD member countries, with reductions in the annual incidence of AUD of 5–14% ^40^. Large scale implementation of advice and treatment programmes can be expensive because of staff and drug costs, but has the potential of large reductions in health care expenditures, with, in some countries, advice and treatment programmes estimated to be cost saving by large margins ^40^. Such programmes would also free large numbers of working age people per year from alcohol-related diseases.

### Increasing PHC activity

Two systematic reviews ^42, 43^ and two multi-country studies ^41, 44, 45^ have demonstrated the possibility of increasing the proportion of patients screened, and screen-positive patients given advice by their PHC providers. The WHO Phase III four-country study on the identification and management of alcohol-related problems in primary care found that the odds ratios for the impact of training and support on increasing higher screening proportions (defined as 20% or more of eligible patients screened) was 2.2 (95% CI=1.3 to 3.1) and on increasing higher intervention proportions (defined as 10% or more of eligible patients screened and advice given to screen positives) was 2.8 (95% CI = 1.6 to 4.0), albeit from very low baseline levels ^45^. In the more recent five-country European ODHIN (Optimizing Delivery of Health Care Interventions) study, providing training and support to PHC providers increased the number of patients screened by 50%, and providing financial reimbursement to PHC providers increased the number of patients screened by 100%, also from low baseline levels of 6/100 consulting adult patients screened ^41^. Other evidence has suggested that the impact of financial incentives on screening and brief alcohol advice in England might have limited effects ^46^. Although incentivised practices recorded higher levels of activity than those not paid to deliver alcohol interventions, overall rates of delivery remained low.

Most work has been undertaken in high-income countries. Whilst there has been some work in low- and middle-income countries ^47^, including countries of Latin America ^48– 53^, there is an opportunity to fast-track scale-up research and practice in such countries. ^54^

### Overcoming constraints on PHC activity

To date, impacts in increasing PHC provider activity have been modest ^55^. There are two important possible reasons for this, which we address in this protocol. The first reason is that standard cut-offs for the frequently used screening instrument, AUDIT-C ^56^ (commonly five for both men and women, or five for men and four for women) to trigger advice are too low, being equivalent to an average daily alcohol consumption of about 20g of alcohol or less ^57^. Practitioners may well find themselves averse to intervening at such low levels, which would also have huge resource implications, with one in three or four patients being eligible for advice. Cut-off points for managing raised blood pressure are commonly determined by levels of blood pressure at which treatment has shown to be effective ^58^. Similarly, cut-off points for brief advice could be the baseline levels of alcohol consumption found in the randomized controlled trials that have investigated the effectiveness of PHC delivered brief advice. In the first Cochrane review of the topic, when reported, baseline levels ranged from 89 to 456g per week, with an overall mean across trials of 313g per week ^59^. At a mean of 313g per week (45g per day, a little lower than the definition of heavy drinking, 50g of alcohol per day, given above), the equivalent AUDIT-C cut off would be 8 ^57^. That lower cut-offs may be inappropriate is also illustrated by the lower effect sizes found in an updated Cochrane review, where the average baseline consumption at enrolment had dropped to 183g/week ^60^. It has also been suggested that PHC providers might be more engaged in screening and giving brief advice, if screening were targeted to patients with comorbid conditions, such as depression or hypertension ^30, 61, 62^. However, to date, there is insufficient evidence for an appropriate package that deals with comorbidity to scale-up ^63^. Further, it has been shown that targeted screening misses out on the vast majority of patients that would be captured by universal screening ^64^. Given the strong associations between harmful alcohol use and depression ^65, 66^, our protocol includes screening for depression and appropriate PHC-based management ^67– 69^ or referral for those patients identified as screen positive by AUDIT-C.

The second reason for modest increases in PHC-based activity could be due to a focus on providers alone, whereas successful implementation of health interventions within complex health system demands addressing a range of underlying structural and support systems ^70^. Phase IV of the WHO study on the identification and management of alcohol-related problems in primary care ^71^, outlined a range of conclusions for enhancing the widespread uptake of screening and brief advice programmes to reduce the harmful use of alcohol: (i) training and practice-based materials need local customization that can be achieved through focus groups; (ii) reframing views about alcohol of both professionals (through training) and the public (through mass media campaigns) is essential; (iii) the establishment of a lead organization is essential, gathering endorsements from a range of organisations and individuals that are highly relevant to the aims of the work; and (iv) adequately controlled community-based studies need to be undertaken to strengthen the evidence base for achieving routine implementation ^71^. The WHO Phase IV study concluded that embedding PHC-based screening and brief advice programmes within the frame of supportive community and municipal environments might lead to improved outcomes. Experience from the US-based SAMHSA SBIRT initiative ^72^ stressed the importance of local champions and whole practice buy in for successful implementation ^73, 74^.

**Figure 1.**
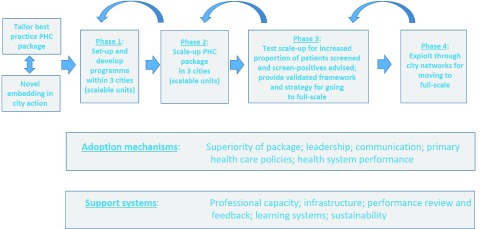
Sequence of activities for going to scale. The four phases of going to scale from setting up the programme within the three cities to exploiting the validated framework and strategy through city networks, with the adoption mechanisms and support systems. PHC, primary health care.

This protocol outlines the design of a quasi-experimental study to test the scale-up of PHC based screening and brief advice programmes to reduce heavy drinking at city level in three Latin American middle-income countries ^75^ (Colombia, Mexico and Peru), in which the prevalence of AUD is 6, 7 and 3%, respectively, and the prevalence of heavy episodic drinking is 4, 11 and 12%, respectively ^4^. We will base our action on the Institute for Healthcare Improvement’s (IHI) framework for ‘going to scale’, which designates four steps in a sequence: (1) *Set-up*, which prepares the ground for introduction and testing of the intervention that will be taken to full scale; (2) *Develop the Scalable Unit*, which is an early testing phase; (3) *Test of Scale-up*, which then tests the intervention in a variety of settings that are likely to represent different contexts that will be encountered at full scale; and (4) *Go to Full Scale*, which unfolds rapidly to enable a larger number of sites or divisions to adopt and/or replicate the intervention ^70^, see Figure 1. We call the proposed study SCALA (Scale-up of Prevention and Management of Alcohol Use Disorder in Latin America).

### Aim and objectives

Driven by implementation science ^70, 76– 83^, this three-country study aims to test the extent to which embedding PHC-based screening and brief advice activity within supportive municipal action leads to improved scale-up of more patients with heavy drinking receiving appropriate advice and treatment. The study has the following objectives:

1. To deliver a tailored package for improving prevention and early identification of heavy drinking, with advice and treatment for case positives that is scalable at municipal level in a wide range of middle- income countries;
2. To set-up and implement the scalable package with key stakeholders in three case study cities (scalable units) from Colombia, Mexico and Peru;
3. To test the scale-up of the package for its impact on provider delivery of early identification and management;
4. To identify and document the facilitators and barriers, and the organizational and resource requirements for going to full-scale, including full economic analyses; and
5. To present a validated framework and strategy for going to full-scale, embedding the package into routine policy and practice, taking into account aspects of stigmatization and equity, that can be replicated globally in the future throughout municipalities.

Our hypothesis is that, by embedding the primary health care action in a community and municipal setting with added support will lead to a greater proportion of patients screened and advised for heavy drinking than achieved hitherto in implementation studies that focused on providers alone.

Countries from Latin America are selected as this is a sub-region of the world in which alcohol jumps from ninth globally to the fourth most important risk factor for morbidity and premature death ^14^. The three specific middle-income countries are chosen to represent Central (Colombia and Mexico) and Andean (Peru) Latin America. The three countries have pre-existing collaboration between the authors, who have experience in the area ^48– 53^.

## Protocol

### Design

**Figure 2.**
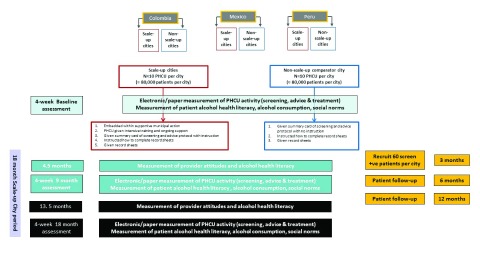
Study design and flow for the three scale-up cities and the three comparator cities, with data gathered during each measurement period. PHCU, primary health care unit.

The study is a quasi-experimental design ^84^, comparing changes in screening and brief advice, and, if relevant, referral for treatment activity, amongst primary health care units (PHCUs) in intervention cities with PHCUs in similar control cities, Figure 2.

### Cities

Intervention municipalities that have confirmed technical and political consent to be involved have been investigator-selected from Bogotá (Colombia), Mexico City (Mexico) and Lima (Peru). Comparator municipalities have been investigator-selected in Bogotá, Mexico City and Lima, on the basis of comparability with the scale-up municipality in terms of socio-economic and other characteristics which impact on drinking, health care and survival, comparable community mental health services, and sufficient geographical separation to minimize spillover effects from the intervention municipality. Randomized selection of the municipalities was excluded as the hypotheses and the study approach relies on municipal-level interventions. Cities are chosen as the scalable unit, as there is a systemic global trend for municipalities to increasingly take on the jurisdictional responsibilities for prevention and health care services. Cities, themselves, are active in prevention and health promotion programmes, and there is a strong evidence base for their impact, also in the prevention of alcohol-related harm ^85, 86^. Cities are a natural site for preventing alcohol-related harm ^87^. Although not having the full jurisdictional responsibilities of national governments for all alcohol policy issues, they often have greater flexibility and are an important site for both media-based and social norms programmes, as well as environmental measures to manage and limit availability of alcohol ^88^. Networks of cities are natural vehicles for exploitation of the results and deployment to full scale, more so with the trends of increasing urbanization in Latin America ^89, 90^.

### Primary health care

Primary care-focused health initiatives can improve access to health care, including among the poor, at reasonably low cost in low- and middle-income countries ^91^, and particularly so in Latin America ^90^. Health-system reforms in Latin America have placed a strong emphasis on the development of comprehensive PHC as a vehicle to achieve universal health coverage, reduce inequities, and democratise health through participation. However, they face ongoing challenges, in particular, the development of health services that can meet the emerging health needs brought on by social and demographic transitions, including the increasing chronic disease burden, and the impacts of rapid urbanisation ^92^.

Management of chronic diseases relies on opportunistic case finding, assessment of risk factors, detection of early disease, identification of high-risk status, combined psychosocial and pharmacological interventions, and long-term follow-up with regular monitoring and promotion of adherence to advice and treatment. Such approaches are financially feasible and have the potential to substantially reduce the burden of chronic diseases. Many interventions can be managed effectively by non-specialists and lay health care workers who are supported by specialists. Although implemented in a range of settings, collaborative care models seem best delivered in PHC settings ^93^. Evidence demonstrates the effectiveness of PHC-based lifestyle interventions in Latin American contexts ^94, 95^, including brief advice programmes to reduce heavy drinking, as well as the potential to detect and refer high-risk patients.

### Participants

Approximately ten PHCUs per intervention and comparator cities will be involved, 60 PHCUs in total. The exact number of PHCUs will depend on the average number of registered patients per PHCU. In each city, the total number of recruited PHCUs should cover a population of about 80,000 registered patients (including children and adults). In jurisdictions, where PHC physicians work as individual practitioners, a PHCU can be defined for the purposes of the study as a virtual or physical location where three or more PHC physicians work. Identification of PHCUs who agree to participate in the studies will be drawn from administrative or academic registries of PHCUs at national, regional, or city levels. The process of recruiting PHCU will be described in detail by each country. Within each PHCU, eligible providers will include any fully trained medical practitioner, nurse or practice assistant with a non-temporary employment contract, working in the PHCU and involved in medical and/or preventive care. These providers will sign an informed consent for their participation. Dependent on customary country practice, participating PHCUs will receive a study fee.

### SCALA care pathway for heavy drinking

The SCALA care pathway includes three integrated components:

i. preventing the development of heavy drinking via increased alcohol health literacy;
ii. screening and brief advice to reduce the prevalence of heavy drinking; and
iii. diagnosis and clinical management of severe AUD and/or co-morbid depression.

The SCALA intervention package deals primarily with the first two parts, prevention and management of heavy drinking. It does not specifically address managing severe AUD, including alcohol-related physical complications and/or severe co-morbid mental health conditions, but ensures the necessary links with specialist services in order to do so, even though specialist treatment can be managed in PHC, with appropriate support ^67, 96^.

Whilst AUDIT-C is highly effective at identifying heavy drinking, it is not designed to stratify patients by severity of AUD, nor designed to diagnose depression, commonly comorbid with heavy drinking. A DSM-5 11-item instrument can be used to stratify the severity of AUD into mild (2–3 items), moderate (4–5 items) and severe (6+ items) ^97^. Similarly, the Patient Health Questionnaire 9 (PHQ-9), can be used to diagnose moderately severe or severe depression with a cut-off score of 15+ ^98^. In our protocol, patients scoring 8+ on AUDIT-C, will be further screened with the DSM-5 11-item instrument and the PHQ-9 to assess severity of AUD and to identify patients with co-morbid depression.

**Figure 3.**
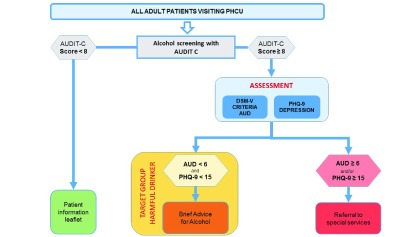
Comprehensive care pathway of SCALA. For screen negative patients, screen positive patients without AUD and depression and for screen positive patients with AUD and/or depression. PHCU, primary health care unit; AUD, alcohol use disorder.

For the care pathway ( Figure 3), all adult patients (age 18+ years) visiting the PHCU for whatever reason will be screened with AUDIT-C, with country-specific pictograms of standard alcohol beverages used to identify the standard unit (drink) of alcohol. Patients with an AUDIT-C score of <8 will be given a patient information leaflet to improve alcohol health literacy (knowledge of the risks of drinking alcohol, and skills to achieve and maintain lower risk drinking, defined as no more than 20g of alcohol per day). Patients with an AUDIT-C score of 8+ will be invited to complete the DSM-5 11-item instrument and the PHQ-9: those with an 11-item score of <6 and a PHQ-9 score of < 15 will be given brief advice of between 5–10 minutes, based on the FRAMES principles ^99^. Those with an 11-item score of 6+ and/or a PHQ-9 score of 15+ will be refereed to more specialist services, at the clinical decision of the health care provider A record of what steps are taken will be recorded on paper or electronic tally sheets prepared for the study.

### Implementation strategies

In the intervention cities, implementation strategies will comprise three components: tailoring the PHC screening and advice package; providing specific practice-based training and ongoing support to PHCUs; and, implementing city-based adoption mechanisms and support systems, including media-based campaigns to improve alcohol health literacy. In the intervention cities, all PHCUs will be given a summary card of screening and advice procedures, with instruction, instruction on how to complete record sheets, and record sheets. In the control cities, all PHCUs will be given a summary card of screening and advice protocol, with no instruction, instruction on how to complete record sheets, and record sheets. As part of the study, no other action will take place in the control cities.

### Tailoring PHC screening and advice package

SCALA is a trans-cultural study, with different health systems, and differences in drinking patterns and attendance at PHC centres, compounded by gender differences. In Mexico, for example, men consume more alcohol then women, but attend PHC services much less frequently than women ^53^. Thus, there is a need for careful tailoring of the screening and advice package. Each intervention city will create Community Advisory Boards (CABs) representing academia, city health and public health departments, health service commissioners and practitioners, and patient and public engagement groups; and User Panels (UPs) of user groups, including PHC providers, patients and citizens. Through expert meetings, workshops, and focus groups, the package will be fine-tuned and tailored to the needs of each city, based on the Tailored Implementation for Chronic Diseases initiative ^100– 102^ within the seven domains of: local and national guideline factors; individual health care provider factors; patient factors; interactions between different professional groups; incentives and resources; capacity for organizational change; and, social, political and legal factors. At the city level, tailoring will be based on the principles of integration between PHC and municipal services ^103^ and the development of complementary community ecosystems that support reductions in heavy drinking. At the PHC level, tailoring will be based on the principles of co-production of health ^104^ between PHC providers and patients.

### Training and ongoing support of PHCUs

In the intervention cities, PHCUs will be offered two initial two-hour face-to-face educational trainings prior to the 18-month scale-up phase, and two one-hour booster sessions during the first twelve months of the 18-month scale-up phase. Training will take place within the PHCU or clusters of PHCUs. Training will be undertaken by peer trainers, members of the research team, accredited teachers, or addiction consultants. Training will focus on management and administrative skills within the primary health care center, on practical skills in undertaking screening and in delivering brief advice, in using the questionnaires, and in knowing when and how to refer patients with more severe AUD ^105– 108^ and moderately severe or severe depression to available services, such as community-based mental health and addiction centers ^67^. Training will, in addition, address attitudes, and perceived barriers and facilitators ^109– 111^ in implementing screening and brief advice, contextualized to local circumstances ^112^. Each country will use an adapted existing country-based training and support package. Where these do not exist, training and support packages will be adapted based on the PHEPA (Primary Health Care European Project on Alcohol) training programme ^113^, widely implemented since 2002 in Catalonia, a Catalan/Spanish speaking, bilingual geographic area. The PHEPA training programme is similar to those used in the WHO Phase III trial ^45^ and the ODHIN study ^41^.

### Implementing city-based adoption mechanisms and support systems

Within each intervention city, an integrator (champion and knowledge and practice broker) will be appointed with responsibilities of serving as a trusted and accountable leader: facilitating agreement within the city and health systems on shared goals and metrics; assessing and acting on relevant community resources; working at the systems level to make relevant practice changes for sustainability; gathering, analyzing, monitoring, integrating, learning, and sharing data at the individual PHCU and city levels; identifying and connecting with system navigators who help PHCUs coordinate, access, and manage multiple services and supports; and developing a system of ongoing and intentional communication with PHCUs and cities.

Within each intervention municipality, the Community Advisory Boards will identify adoption mechanisms that can be used for scale-up, for example: (i) demonstration of the superiority of the PHC package, its simplicity, and its alignment with the latest evidence of preventing and managing heavy drinking and of implementation science; (ii) engagement of identified leaders and building their capacity to lead and ensure broad adoption of the PHC package through guiding and supporting large-scale change ^114– 116^; (iii) communicating the value of the PHC package to both municipal and PHC frontline staff ^117^; (iv) identifying and adjusting, as appropriate and possible, relevant policies at PHC and city levels to expedite the adoption of the PHC package, for example by adapting electronic health records; and, (v) identifying gaps in health system performance and the urgent need to prevent and manage heavy drinking to promote the needed will and energy to bring implementation of the PHC package to scale ^118^.

The Community Advisory Boards will also identify additional mechanisms that can be used to support scale-up, for example: (i) development of professional capacity for scale-up; (ii) development of infrastructure for scale-up, achieved through redesign rather than addition of new resources; (iii) linking to monitoring and evaluation, using reliable data collection and reporting systems that track and provide feedback on the performance of key processes and outcomes, for example monthly reporting on screening and brief advice activity; (iv) setting up learning systems to capture change ideas that are shown to result in improved performance assembling ideas into a change package. Knowledge should be shared between municipal actors and PHCUs through regular electronic newsletters and communications ^119^; and, (v) creating design factors that enhance sustainability including high reliability of the new processes, inspection systems to ensure desired results are being achieved, support for structural elements, and ongoing learning systems ^120, 121^.

### Data collection

Based on the validated methodology of the ODHIN project ^41, 122^, PHC providers will document activity by completing paper or electronic (depending on the ability to use existing electronic health records) anonymous tally sheets that record eligible patients’ (aged 18+ years) AUDIT-C scores, if administered, DSM-5 11-item and PHQ-9 scores, and the advice or treatment given to each patient. The tally sheets will record the age, sex, employment status, and educational level of the patient, the latter as one proxy measure of socio-economic status. The tally sheets will also include: two questions that capture previous experience of being asked about how much the patient drinks and of being advised to reduce the amount drunk to provide information for UN Sustainable Development Goal 3.5 ^12^; one question about alcohol being a cause of high blood pressure, liver problems, depression or cancer, as a simple measure of alcohol health literacy (knowledge part) ^123^; and, two questions about injunctive social norms of drinking alcohol ^124^.

Data will be collected for each calendar month during the 18-month scale-up period. Formal evaluation will take place during three measurement periods: 4-week baseline period; 4-week assessment period during the 9 ^th^ month of the 18-month scale-up period; and, 4-week assessment period at 18-months, the end of scale-up period. PHCUs will return data on the number of adult (aged 18+ years) consultations per provider for the four-week baseline assessment period, and for each of the 18 months of the scale-up period.

At baseline, PHC providers will provide data on their age, sex and profession (doctor, nurse, practice assistant etc.). At baseline, and at two time points during the 18-month scale-up period (month 4.5 and month 13.5), providers will provide data on their alcohol health literacy and on their attitudes to working with patients with heavy drinking. The alcohol health literacy instrument will assess knowledge of risks due to drinking ^123^, and descriptive and injunctive social norms ^124^. The attitudes instrument will be the shortened version of the Alcohol and Alcohol Problems Perception questionnaire ^125^.

During month 3 of the 18-month implementation period, the first six consecutive screen positive patients identified by each PHC provider will be invited to give their consent to complete two follow-up questionnaires, at six months and twelve months after the initial screening. The patient interviews will be used for quality control ^126^, but not as a study outcome measure. The follow-up questionnaires will be the same as the baseline questionnaire and will be undertaken by the local academic unit by face-to-face or telephone interview. Collected data will include sex, age, educational level, alcohol consumption (operationalized by AUDIT-C), alcohol health literacy, prevalence of depressive symptoms using the nine-item patient health questionnaire ^98^, experience of screening and brief advice and treatment for heavy drinking, experience of self- and co-management for heavy drinking and health service utilization.

Process evaluation will be ongoing through interviews with CABs, with formal evaluation time points at baseline, ninth month of the 18-month scale-up period, and at the end of the scale-up period. Logic models will be developed and data will be collected on drivers, facilitators and barriers of successful implementation ^127, 128^. City and country-based contextual, financial and political-economy factors will be collected (see outcomes below).

During all phases of the scale-up, we will document impact on other sectors (education, social care, criminal and justice, etc.) based on resource use measurement ^129^. Patients in the scale-up and comparator cities will be asked to complete a short questionnaire about resource use measurement. Costs will be calculated by multiplying volumes (resource use) with unit costs, based on guideline prices ^130^. Health and disability will be measured by the WHO Disability Assessment Schedule (WHODAS 2.0) ^131, 132^. QALYs will be derived through transformation of the WHODAS 2.0 12-item scores. A probabilistic Markov decision analytic model will be built in to estimate the expected cost per outcome and the costs per QALY of SCALA from a societal perspective, based on established economic evaluation state-transition modelling guidelines ^133, 134^. Costs and effects will be modelled for five years and life time. Probabilistic sensitivity analyses will be executed.

All relevant data required for testing the scale-up will be transferred to the institution leading the evaluation work (Technische Universitaet Dresden) in accordance with its research data protocols. No individual data will be published, and data will only appear in aggregate form in project publications. On publication of the results, datasets will be made available via the UK data archive service ( http://www.data-archive.ac.uk/).

### Outcomes

**Primary outcome:** The primary outcome will be the proportion of consulting adult patients intervened (screened and advice given to screen positives), calculated as the number of AUDIT-C positive patients that received oral advice or referral for advice to another provider in or outside the PHCU, divided by the total number of adult consultations of the participating providers per provider and per PHCU.

**Secondary outcomes:**

**-**  **Screening and advice:** The proportion of patients screened will be calculated as the number of completed screens divided by the total number of consultations of all patients eligible for screening (as defined above) per participating provider, and averaged per participating PHCU. The proportion of patients advised will be calculated as the number of brief interventions delivered (received oral brief advice, and/or were given an advice leaflet, and/or were referred to another provider in or outside the practice), divided by the total number of screen positives per participating provider and averaged per participating PHCU. Information will also be collected on the number of screen negatives who received brief advice.
**-**  **Provider attitudes and provider alcohol health literacy:** Attitudes of the participating providers will be measured by the short version of the Alcohol and Alcohol Problems Perception questionnaire, SAAPPQ ^25, 135– 137^. The responses will be summed within the two scales of role security and therapeutic commitment. Individual missing values for any of the items in a domain will be assigned the mean value of the remaining items of the domain before summation. Provider alcohol health literacy will be assessed through knowledge of risks due to drinking ^123^, and reported descriptive and injunctive social norms of drinking ^124^.
**-**  **Patient alcohol health literacy and injunctive social norms:** the tally sheets include one question about alcohol being a cause of high blood pressure, liver problems, depression or cancer, as a simple measure of alcohol health literacy (knowledge part) ^123^; and, two questions about injunctive social norms of drinking alcohol ^124^. We will analyze changes over time from baseline to the end of the 18-month implementation period, comparing changes in the screened population of alcohol health literacy and injunctive social norms between intervention and control cities.

### Process measures

**Figure 4.**
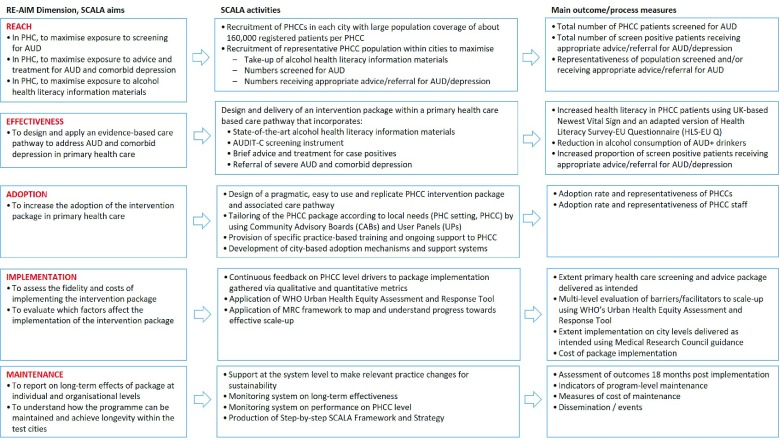
RE-AIM dimension and SCALA aims, activities and main outcome/process measures. PHCU, primary health care unit; PHC, primary health care; AUD, alcohol use disorder.

We will use the RE-AIM Framework as our basis to evaluate SCALA’s impact across the five dimensions of reach, efficacy, adoption, implementation, and maintenance ^138– 140^, ensuring fidelity in its completion ^141^, Figure 4.

At least four elements will be included. First, a driver diagram ^142^ will be used to identify drivers for successful scale-up. To enable a nuanced understanding of how scale-up varies in the different cities, recognizing that context can have a greater influence on scale-up than any pre-specified implementation strategy, the driver diagrams will provide real- time continuous feedback on how changing contexts in health systems or city actions affect outcomes. Second, the evaluation procedure of WHO’s Urban Health Equity Assessment and Response Tool (Urban HEART) ^143^ will be modified to identify the barriers and facilitators to scale-up. Third, the factors influencing the progress from scale-up to outcomes will be identified and documented based on UK Medical Research Council guidance ^144^ analysing factors within five groups: (i) description of intervention and its causal assumptions; (ii) context; (iii) implementation; (iv) mechanisms of impact; and, (v) outcomes. Fourth, using the detailed methodology of Ysa *et al.* ^145^, the experience and outcomes of the scale-up will be mapped with contextual, financial and political-economy analyses of the cities and the countries within which they are located. The following contextual factors will be collected: (1) available data similar to that of the OECD better life initiative ^146^, including material living conditions (housing, income and jobs) and quality of life (community, education, environment, governance, health, life satisfaction, safety and work-life balance); (2) Sustainable Governance Indicators ^147^, including the Status Index, which ‘examines each state’s reform needs in terms of the quality of democracy and performance in key policy fields’, and the Management Index, focused on ‘governance capacities in terms of steering capability and accountability’; and, (3) World Values Survey data ^148, 149^ for cross-cultural variation (Traditional vs. Secular-rational; and, Survival vs. Self-expression). Documentation will be complied either at municipal or country level for alcohol policy-related strategies, action plans, legislation and evaluations. A model will be built on two levels of analyses, contextual factors and policy factors and this will be mapped on to the test of the scale-up of the PHC interventions to describe and identify those contextual and policy factors that might influence going to full-scale beyond the implementation cities.

### Sample size

Our power calculations are based on the following assumptions: at baseline, 2.5/1,000 consulting patients will be found to be screen positive (based on an AUDIT-C cut-off score of 8) and advised to reduce their alcohol consumption (data from ODHIN study; Anderson, personal communication). To detect an increase in the number to 5/1,000 (a doubling), with 80% power and a significance level of 5%, and assuming a design effect of ten PHCUs per three cities per group (scale-up and comparator), with an ICC for PHCUs across countries = 0.03 (data from ODHIN study; Anderson, personal communication), a conservative estimate of 30 PHCUs across three scale-up cities and 30 across three comparator cities, about ten per city will be needed ^150^, assuming an average PHCU size of about 8,000 patients with a monthly consultation rate of 1,200 adult patients per PHCU (data from ODHIN study; Anderson, personal communication).

### Statistical measures

The primary outcome of the study will be the proportion of consulting adult patients intervened (screened and advice given to screen positives) measured during two four-week periods midway and at the end of the 18-month scale-up period, and this will be analysed at the levels of the PHCU and provider by city type (intervention or control) ^151^. Given the rarity of the event and the resulting distribution, we will use exact inference methods for comparison of intervention vs. control cities. For further analyses, including covariates, regression models will be used, taking into consideration the hierarchical nature of the data ^152^, and characteristics at different hierarchy levels (i.e., characteristics of the PHCU, characteristics at the city level, such as patterns of drinking), and incorporating 4-week baseline period measurements as covariates. Special consideration will be given to the skewness of data by applying models, such as zero-inflated binomial regression, after testing for necessary assumptions ^153, 154^. Odds ratios will be presented with 95% confidence intervals. For any PHCU or provider that drops out during the study, outcome values for subsequent measurement points will be set at the last value obtained.

### Ethics

Before any involvement of participants in the study, including patients consulting in the study PHC units, the respective country-based partner in Colombia, Mexico and Peru will comply with their national legislation, regulations and ethical principles by applying for an ethical approval for research at the competent ethical authorities in their jurisdiction.

## Discussion

This protocol outlines a quasi-experimental study ^84^ to test the extent to which embedding PHC-based screening and brief advice activity within supportive municipal action leads to improved scale-up of more patients with heavy drinking receiving appropriate advice and treatment.

For a wide range of health care issues, including communicable and non-communicable diseases, as well as reproductive and child health care, major variations continue to exist in many dimensions of quality of care, including safety, efficiency, effectiveness, timeliness, patient centeredness, and equity ^70^. This can be understood as a failure to equitably scale up excellent care to ensure that what we know works is delivered to everyone who needs it.

There is a wealth of literature on implementation science and quality improvement, and a range of frameworks exist that include a sequential approach for scale-up, and that provide practical guidance for how to work with organizations, health systems, and communities to implement and scale-up best practices ^76– 83^.

In choosing a framework to adopt and apply, we wanted one that draws together: the main themes of sequencing activities to get a complex health system intervention, with elements of prevention and management, to full scale; the mechanisms that are required to facilitate the adoption of a complex health system intervention; and, the underlying factors and support systems required for successful scale-up. We also wanted a framework that includes a scalable unit at meso- (in our case city) level that provides the key infrastructural components and relationship architecture that are likely to be common across cities that are part of networks, (e.g., Healthy Cities Networks) enabling a more likely successful transition to full scale.

A key framework that meets all these needs is that of the Institute for Healthcare Improvement (IHI) which identifies adoption mechanisms and support systems for use across the steps, and identifies the implementation methods that can be used at each step, that we have incorporated into our protocol ^70^.

The proposed study has several features that merit attention.

First, we simplify and account for cultural differences in definitions of AUD ^18, 19^, by using heavy drinking ^20, 21^ as our operational approach, rather than AUD or harmful use of alcohol ^1– 3^.

Second, we set a higher cut-off score for AUDIT-C (8+) than is commonly used to classify screened case-positives, matching definitions of heavy drinking ^23, 24^, and similar to baseline levels of alcohol consumption in PHC-based trials to reduce heavy drinking ^59^. We also set the same cut-offs for men and women, based on epidemiological evidence ^24^, and minimizing unintended consequences of using different cut offs for men and women ^155^.

Third, we limit brief advice to 5-10 minutes, rather than using more intensive interventions ^67^, since the evidence suggests that brief advice is as effective and cost-effective as more extended advice or treatment in reducing heavy drinking ^33, 34, 156, 157^.

Fourth, we recognize the importance of comorbid moderately severe and severe depression ^65, 66^, by building in identification and referral mechanisms, recognizing that moderately severe and severe depression can be well-managed with sufficient support systems in PHC ^67– 69^.

Fifth, based on evidence ^71^, we adopt a novel approach by embedding and scaling-up the PHC activity within cities, supported by a series of city-based adoption mechanisms and support systems ^70^, and enhanced alcohol health literacy ^158^, aiming to assist in building a new knowledge base, on which better policy could be based.

Sixth, we use a theory-based approach to tailoring ^100– 102^, creating city-based Community Advisory Boards, and user-based UPs to ensure that tailoring matches user needs, municipal services ^103^, and co-production of health ^93, 104^.

Seventh, we include a range of outcome measures, including patient outcomes, as a quality check ^126^, which address weaknesses of many previous implementation studies in this area, which have focussed on provider outcomes, rather than patient outcomes ^42, 43^. Through the use of existing electronic health records, and further to ethical and confidentiality agreements, we anticipate the ability to link individual AUDIT-C scores with consultations within the primary health care centers and hospitalizations within district hospitals, recording diagnosis for both fully and partially attributable alcohol-related conditions ^13^.

Eighth, we have a longer time frame (18 months) than is traditionally used in implementation studies ^41, 44, 45, 159^, to assess longer term impacts.

Ninth, we give considerable emphasis to process evaluation ^144^, developing logic models to document the fidelity of all implementation strategies, and to identify, the drivers and barriers and facilitators to successful implementation and scale-up, and the political and economic contextual factors that might influence scale-up, based on the RE-AIM framework ^138^.

And, finally, tenth, we place the study design in the public domain, so that others might replicate the study approach (with acknowledgment) to see if the scale-up principles can work across jurisdictions. In so doing, we would be pleased to receive comment and feedback.

We are aware of some limitations of the study design. As we are unable to randomize the involved cities, we adopt a quasi-experimental design, recognizing that it is not possible to randomly allocate the municipalities. Randomized selection of the municipalities was excluded as the hypotheses and the study approach relies on municipal-level interventions. A trial with random assignments of municipalities is not feasible due to cost (number of municipalities) and municipal-based political and technical considerations. Randomization of primary health care centers within municipalities is also impossible for the same reasons of municipal involvement in the interventions. Clean control conditions in this environment where the municipality supports primary health care-based does not seem to be possible. As a result, we created a quasi-experimental design ^84, 160, 161^, trying to optimize control for confounding, and using propensity score matching (PSM), given the above constraints. While full control via randomization, and thus establishment of causality is not possible, together with the qualitative evaluation component of the study, we will be able to clearly identify the mechanisms which were crucial in leading to the outcomes. According to a recent 7-item checklist for classifying quasi-experimental studies for Cochrane reviews ^162^, our approach is, nevertheless, ranked as a strong design.

Although our focus on embedding PHC activity within supportive municipal actions is hypothesized to increase screening and brief activity over and above that previously demonstrated, such an approach also brings risks. Municipal governments change; and, thus health priorities may change. Although our approach minimizes the need for extra resources (and in some jurisdictions, could be resource saving ^34, 40^), it is not resource free. Funding constraints could limit future scale-up and sustainability.

We have adopted two approaches to promote sustainability. First, our protocol is based on transdisciplinary research, which is an approach that: identifies, structures, analyses, and deals with specific problems in such a way to grasp the complexity of problems ^163^; takes into account the diversity of life-world and scientific perceptions of problems; links abstract and case-specific knowledge; and, develops knowledge and practices that promote what is perceived to be the common good ^164^. As such, we involve municipalities as stakeholders to form explicitly orchestrated and managed ecosystems that cross organizational boundaries. Municipalities will create an appropriate engagement platform that provides the necessary environment, including people and resources, for sustainability. Second, we have chosen municipalities as the level of scale, making use of the existing Latin American and Caribbean (LAC) Healthy Cities Network as a natural platform for going to full-scale.
