# Supplementary material for: Scaling-up primary health care-based prevention and management of heavy drinking at the municipal level in middle-income countries in Latin America: Background and protocol for a three-country quasi-experimental study
Source: F1000Res. 2017 Nov 13;6:311. Originally published 2017 Mar 23. [Version 3] doi: 10.12688/f1000research.11173.3 (PMC5686480; doi:10.12688/f1000research.11173.3)
Supplement: Peer review history

# Peer review: Referee response for version 3

**DOI:** 10.5256/f1000research.14303.r27883

**Published:** 2017-11-20

**Authors:** John B. Saunders

**Affiliations:** 1 Centre for Youth Substance Abuse Research, University of Queensland, Brisbane, QLD, Australia

I am happy to approve without any further amendments Version 3 of this paper for publication.

I have read this submission. I believe that I have an appropriate level of expertise to confirm that it is of an acceptable scientific standard.

# Peer review: Referee response for version 3

**DOI:** 10.5256/f1000research.14303.r27884

**Published:** 2017-11-13

**Authors:** Janna Skagerström

**Affiliations:** 1 Research and Development Unit in Region Östergötland, Department of Medical and Health Sciences, Linköping University, Linköping, Sweden

Thanks for your anwers and changes in the manuscript. I have no further comments to make.

I have read this submission. I believe that I have an appropriate level of expertise to confirm that it is of an acceptable scientific standard.

# Peer review: Referee response for version 2

**DOI:** 10.5256/f1000research.13245.r27075

**Published:** 2017-11-02

**Authors:** Janna Skagerström

**Affiliations:** 1 Research and Development Unit in Region Östergötland, Department of Medical and Health Sciences, Linköping University, Linköping, Sweden

Thanks for a well written protocol of an interesting study.

 I have a few questions concerning the protocol part of the manuscript that needs to be addressed.

 In Figure 2 it is stated that patient alcohol health literacy is going to be measured at baseline and 18-month assessment. Further, on page 8 it is stated that the intervention cities will have media-based campaigns to improve alcohol health literacy. However, I can’t find any outcomes related to the patient (or city-inhabitant) alcohol health literacy. Please elaborate on how the data gathered from patients on Health literacy and social norms will be used.

 Is the number of AUDIT-C positive inhabitants expected to be the same in all catchment areas at baseline? If not, how is this handled in the primary outcome measure?

 Minor details:

 In Figure 4. REACH, in the first bullet point it should be clear that it is exposure to screening for AUD *within PHC.*

 Page 11, 6 ^th^ paragraph of the discussion says “the proposed study has several features than merit attention” should be changed to “..that merit…”

I have read this submission. I believe that I have an appropriate level of expertise to confirm that it is of an acceptable scientific standard, however I have significant reservations, as outlined above.

# Response

**Published:** 2017-11-09

**Authors:** Peter Anderson

**Affiliations:** Newcastle University , UK

In Figure 2 it is stated that patient alcohol health literacy is going to be measured at baseline and 18-month assessment. Further, on page 8 it is stated that the intervention cities will have media-based campaigns to improve alcohol health literacy. However, I can’t find any outcomes related to the patient (or city-inhabitant) alcohol health literacy. Please elaborate on how the data gathered from patients on Health literacy and social norms will be used.

RESPONSE: Thank you for pointing this out. The tally sheets include one question about alcohol being a cause of high blood pressure, liver problems, depression or cancer, as a simple measure of alcohol health literacy (knowledge part); and, two questions about injunctive social norms of drinking alcohol. We will use this information to describe the level of alcohol health literacy and injunctive social norms in the screened population, relating the responses to demographic information and AUDIT-C results. The information will also inform the content of the brief advice delivered to screen positive patients. We had not considered using this data as an outcome of the trial. However, in the intervention cities, we might hypothesize that these measures improve over time in the population of screened patients as compared with the control cities. We have added this as a secondary outcome measure.

Is the number of AUDIT-C positive inhabitants expected to be the same in all catchment areas at baseline? If not, how is this handled in the primary outcome measure?

RESPONSE: In the statistical measures section, we mention that the regression models include the baseline period measurements (for the primary outcome measure, proportions) as a covariate. This together with accounting for the hierarchical nature of the data (multi-level analyses) will handle the issue of any differences in the proportion of screen-positive patients between the different catchment areas.

Minor details:

In Figure 4. REACH, in the first bullet point it should be clear that it is exposure to screening for AUD *within PHC.*

RESPONSE: We have added ‘In PHC’ to the three bullet points in the REACH box. 

Page 11, 6 ^th^ paragraph of the discussion says “the proposed study has several features than merit attention” should be changed to “..that merit…”

RESPONSE: We have corrected.

# Peer review: Referee response for version 2

**DOI:** 10.5256/f1000research.13245.r24613

**Published:** 2017-09-18

**Authors:** John B. Saunders

**Affiliations:** 1 Centre for Youth Substance Abuse Research, University of Queensland, Brisbane, QLD, Australia

**1. Reviewer’s abbreviated comment on original version**

***The introduction starts awkwardly with a description of the alternative ways of conceptualising alcohol misuse. For the definitive version of the paper, I would strongly suggest that the relevant sections of the introduction be reshaped.***

**Authors’ response**

 The nomenclature describing ‘harmful use of alcohol’ and ‘alcohol use disorders’ are cumbersome and somewhat contradictory across organizations and publications, with, for example...

**Reviewer’s comments on this response**

 The response from the authors has missed the point of my comments. Essentially I felt that much of the introduction was irrelevant to the main thrust of the paper and the research proposed within it, and therefore it should be reshaped. The title itself may be misleading because it refers to “alcohol use disorder”. The essential aim of the study is the implementation of interventions for excessive (or hazardous) alcohol consumption or as the authors term it “heavy drinking over time” and it is operationalised as 50+ grams of alcohol per day and an AUDIT-C score of 8+.

 To use “heavy drinking” as the target for the intervention is entirely reasonable and I agree that it fits with epidemiological data and a primary care perspective. My point is that this should be argued for and defined at the beginning of the introduction, eliminating unnecessary material in the first two paragraphs and elsewhere. The title of the study needs to be changed too. The term “alcohol use disorder” is not synonymous with “heavy drinking”. In the Oxford dictionary a disorder is defined as “an illness that disrupts normal physical and mental functions” and the ICD and DSM definitions refer to cognitive, behavioural, and patho-physiological phenomena, with no reference to the amount of alcohol consumed (indeed, in the DSM-5 IRT analyses, consumption variables did not explain any additional variance and so were excluded). I note that patients who have an alcohol use disorder (defined as AUD of 6+) will be referred to specialist services (as is appropriate), rather than being managed as part of the brief intervention. 

 All this is needed is some further reshaping of the introduction and to consider whether the title should be modified to clarify the target of the study.

 The authors may wish to refer to the WHO definition of “hazardous alcohol use” in the draft ICD-11 [This was included in the draft of ICD 10 but it was deleted from the published version because the ICD 10 Committee did not consider it was a disorder and therefore its inclusion could not be justified]. 

**2. Reviewer’s abbreviated comment on original version**

***With regard to the “treatment gap” caution is advised about extrapolating too much from the findings***

**Authors’ response**

 Yes, this is likely true. Nevertheless, there is an apparent and widespread gap between those who might benefit from advice and treatment and those who get it (like high blood pressure). We have added some words of caution in measuring and reporting treatment gaps.

**Reviewer’s comments on this response**

 It is true – check the NESARC data. I entirely accept the rationale for trying to bridge the treatment gap but on average (based on the countries from which we have data on this), the patients in the present study are likely to have a lower level of heavy drinking and fewer comorbid disorders than those who access treatment currently.  

**3. Reviewer’s abbreviated comment on original version**

***Is there any work reported for the three countries selected on the professional supports available to PHC staff?***

**Authors’ response**

 There have been existing studies on testing the impact and implementation of primary health care based screening and advice programmes in Latin American countries, and these are referenced in the paper...

**Reviewer’s comments on this response**

 The revisions address this comment.

**4. Reviewer’s abbreviated comment on original version**

***Reference is made to various scaling-up approaches but the paper would be strengthened by a description and discussion of the theoretical concepts underlying the framework proposed.***

**Authors’ response**

 We have added text on implementation and scale-up literature to the first part of the discussion.

**Reviewer’s comments on this response**

 The revisions address this comment.

**5. Reviewer’s abbreviated comment on original version**

***Will the investigators have access to medical records and PHC throughput data?***

**Author's response**

 In all three countries, electronic health records are used. Provided...

**Reviewer’s comments on this response**

 The revisions address this comment.

**6. Reviewer’s abbreviated comment on original version**

***Overall, this is a very ambitious and complex research program and I became concerned in reading it - in all its complexity - that it might not be achievable with staff who have no prior involvement in research and who are working in resource-constrained health care systems.***

**Authors’ response**

 We respond by considering that these are false and prejudicial assumptions. The CVs and experiences of the investigators in the three country sites surpass those of many investigators who have implemented similar international studies in ‘high-income’ countries. Further...

**Reviewer’s comments on this response**

 Not a bit of it! My comments have nothing to do with the calibre of the investigators or the countries involved. The investigators include some of the most renowned researchers in this field and several also have a detailed knowledge of and working experience in the health care systems of Central and South America.

 I use the term “resource constrained” as referring to competing priorities when there is finite amount of money. Many countries in the middle or lower income bracket have well developed primary health care systems. Two examples are Cuba and Iran. In Iran there is a primary health care network with doctors, nurses and other health care professionals which extend throughout the country and covers more than 98% of the population. I do not have direct knowledge of health care systems of the three countries involved in the study (my experience is mainly in Asian countries), but World Bank reports on the health care systems describe these resource constraints. The response I was expected was something like the following:

 “Country A has a population of X million and a per capita GDP of Y. It has a well developed primary health care system which is based on universal health insurance and provides free health care at the point of contact (or provides reimbursement with minimal or no out-of-pocket costs). This system of primary care covers the entire country apart from certain remote areas and includes 95% of the population. The municipalities selected all have access to primary health care facilities, and … ”.  Information about the municipalities is provided in the protocol.

 This sort of information provides me with confidence that the study proposed is feasible in a particular country. A principle of a paper is that the methods contain sufficient information that (i) allows the reviewer to be confident that the study’s objectives can be met, and (ii) that there is sufficient information that another group of investigators could if they wish replicate the study. I am confident in the authors’ capabilities but that was not the point that I was raising.  

**7. Reviewer’s abbreviated comment on original version**

***There is a statement “During all phases of the scale-up, we will document impact on other sectors (education, social care, criminal and justice, etc.) base on resource use measurement.”  This is a huge additional amount of work.***

**Authors’ response**

 This is a misunderstanding. It requires quite minimal data collection from samples of patients. There are then robust tried and tested methodologies to estimate costs per outcome based on the collected data...

**Reviewer’s comments on this response**

 More information has now been provided which reassures me that what appeared to be a very major and potentially time consuming aim of accessing multiple alcohol and other related social outcomes will, through the availability of linked databases be feasible, in part through a sampling process. 

**8. Reviewer’s abbreviated comment on original version**

***The paper would be more convincing (to this reviewer) if it were linked to budgetary inputs.***

**Authors’ response**

 The purpose of a protocol is to describe the scientific approach. We would not be proposing this study if we did not consider that we had sufficient resources to implement it.

**Reviewer’s comments on this response**

 I would hope not, but the onus is also to provide sufficient information to convince an independent reviewer that the study is feasible. For all the grant giving organisations I know this is essential and it would be a strength of a protocol paper if there was reassurance that the level of support was sufficient for each site to undertake and facilitate the work – in terms of personnel required; I am not asking for the monetary amounts. Again the principle is of providing sufficient information that the study could be replicated if desired by independent investigators after it had been completed. 

**9. Reviewer’s abbreviated comment on original version**

***Although there are approximately 160 references, an impressive number for a protocol paper, there are some surprising omissions***

**Authors’ response**

 We have included references to the WHO Phase III and IV studies, which the first author of this paper coordinated whilst he worked with WHO. We have added the Funk et al. reference (REF 159).

**Reviewer’s comments on this response**

 The revisions address this comment.

I have read this submission. I believe that I have an appropriate level of expertise to confirm that it is of an acceptable scientific standard, however I have significant reservations, as outlined above.

# Response

**Published:** 2017-11-09

**Authors:** Peter Anderson

**Affiliations:** Newcastle University , UK

**1. Reviewer’s abbreviated comment on original version**

***The introduction starts awkwardly with a description of the alternative ways of conceptualising alcohol misuse. For the definitive version of the paper, I would strongly suggest that the relevant sections of the introduction be reshaped.***

**Authors’ response**

The nomenclature describing ‘harmful use of alcohol’ and ‘alcohol use disorders’ are cumbersome and somewhat contradictory across organizations and publications, with, for example...

**Reviewer’s comments on this response**

The response from the authors has missed the point of my comments. Essentially... 

**AUTHOR RESPONSE**: Thank you. You are correct in pointing out that the title could be modified to better reflect the study, and that the introduction, as written, confuses rather than clarifies, and is not necessarily relevant to the study in question. We have modified the title, and shortened the introduction. 

**2. Reviewer’s abbreviated comment on original version**

***With regard to the “treatment gap” caution is advised about extrapolating too much from the findings***

**Authors’ response**

Yes, this is likely true. Nevertheless...

**Reviewer’s comments on this response**

It is true – check the NESARC data. I entirely accept the rationale for...

**AUTHOR RESPONSE** Thank you.

**3. Reviewer’s abbreviated comment on original version**

***Is there any work reported for the three countries selected on the professional supports available to PHC staff?***

**Authors’ response**

There have been existing studies on testing the impact and implementation of primary health care based screening and advice programmes in Latin American countries, and... 

**Reviewer’s comments on this response**

The revisions address this comment.

**AUTHOR RESPONSE** Thank you.

**4. Reviewer’s abbreviated comment on original version**

***Reference is made to various scaling-up approaches but the paper would be strengthened by a description and discussion of the theoretical concepts underlying the framework proposed.***

**Authors’ response**

We have added text on implementation and scale-up literature to the first part of the discussion.

**Reviewer’s comments on this response**

The revisions address this comment.

**AUTHOR RESPONSE** Thank you.

**5. Reviewer’s abbreviated comment on original version**

***Will the investigators have access to medical records and PHC throughput data?***

In all three countries, electronic health records are used. Provided ethical and confidentiality agreements...

**Reviewer’s comments on this response**

The revisions address this comment.

**AUTHOR RESPONSE** Thank you.

**6. Reviewer’s abbreviated comment on original version**

***Overall, this is a very ambitious and complex research program and I became concerned in reading it - in all its complexity - that it might not be achievable with staff who have no prior involvement in research and who are working in resource-constrained health care systems.***

**Authors’ response**

We respond by considering that these are false and prejudicial assumptions. The CVs and experiences of the investigators...

**Reviewer’s comments on this response**

Not a bit of it! My comments have nothing to do with the calibre of the investigators or the countries involved. The investigators include...

**AUTHOR RESPONSE** Our apologies for misunderstanding your original comment. Thank you for clarifying the comment. We withdraw the first sentence of our original response.  The study is now funded by the Horizon 2020 programme of the European Commission.

**7. Reviewer’s abbreviated comment on original version**

***There is a statement “During all phases of the scale-up, we will document impact on other sectors (education, social care, criminal and justice, etc.) base on resource use measurement.”  This is a huge additional amount of work.***

**Authors’ response**

This is a misunderstanding. It requires quite minimal data collection...

**Reviewer’s comments on this response**

More information has now been provided which...

**AUTHOR RESPONSE** Thank you.

**8. Reviewer’s abbreviated comment on original version**

***The paper would be more convincing (to this reviewer) if it were linked to budgetary inputs.***

**Authors’ response**

The purpose of a protocol is to describe the scientific approach. We would not be proposing this study if we did not consider that we had sufficient resources to implement it.

**Reviewer’s comments on this response**

I would hope not, but the onus is also to provide sufficient information to convince an independent reviewer that the study is feasible. For all the grant giving organisations...

**AUTHOR RESPONSE** Thank you. The study is now funded by the Horizon 2020 programme of the European Commission.

**9. Reviewer’s abbreviated comment on original version**

***Although there are approximately 160 references, an impressive number for a protocol paper, there are some surprising omissions***

**Authors’ response**

We have included references to the WHO Phase III and IV studies, which the first author of this paper coordinated whilst he worked with WHO. We have added the Funk et al. reference (REF 159).

**Reviewer’s comments on this response**

The revisions address this comment.

**AUTHOR RESPONSE** Thank you.

# Peer review: Referee response for version 1

**DOI:** 10.5256/f1000research.12056.r23258

**Published:** 2017-06-26

**Authors:** Peter Nygaard

**Affiliations:** 1 Prevention Research Center, Pacific Institute for Research and Evaluation, Oakland, CA, USA; 2 California State University East Bay, Hayward, CA, USA

This is a very important and ambitious study that focuses on some of the known obstacles of implementing screening and brief intervention in primary health care (PHC). However, whereas some of the inherent problems in the procedures are being tested, others remain untouched. For example, it is quite unclear how the authors intend to maintain the interventions after the project ends. It is well-known that you can bring GPs to test SBI interventions but once the projects are over, they return to practice as before. Below are a few points that came to mind reading the article.

 It is quite unclear what the focus of this study is because the primary outcome is basically a rise in patients screened and given advice. It should be evident that this number will go up in participating PHCs because of the intervention per se???

 The process evaluation is definitely a strength of this study because it is comprehensive with a very clear framework of understanding.

 It is difficult to see the advantage of involving three different countries with only one intervention city and one control city from each. As pointed out, there are differences among the participating countries that will have to be accounted for and that will limit the robustness of the results. Furthermore, it is unclear how the participating cities will be chosen and how the intervention city will be chosen. Including three different “cultures” with only one intervention and one control site for each does not seem to be a robust design.

 There should be more considerations about the differences among the participating countries and the impact on the final outcomes. This is a very important study that focuses on some of the known obstacles of implementing screening and brief intervention in primary health care (PHC). However, whereas some of the inherent problems in the procedures are being tested, others remain untouched. For example, it is quite unclear how the authors intend to maintain the interventions after the project ends. It is well-known that you can bring GPs to test SBI interventions but once the projects are over, they return to practice as before.

I have read this submission. I believe that I have an appropriate level of expertise to confirm that it is of an acceptable scientific standard, however I have significant reservations, as outlined above.

# Response

**Published:** 2017-07-24

**Authors:** Peter Anderson

**Affiliations:** Newcastle University , UK

**It is quite unclear how the authors intend to maintain the interventions after the project ends.**

The last major international study in this area, the Phase IV WHO study, referenced in the paper, concluded that embedding primary health care-based screening and brief advice programmes within the frame of supportive community and municipal environments might lead to improved outcomes, and called for adequately controlled community-based studies to be undertaken to strengthen the evidence base for achieving routine implementation. The present study aims to do that -  we have clarified this by adding a hypothesis. It is not completely correct to say that once projects are over, GPs return to practice as before. In the ODHIN study, six months after the completion of the implementation period and the brief training received, providers who had received training were still screening and advising more patients than those who had not received training. It is not the purpose of the study to maintain the interventions after the project ends - nevertheless, we have added text at the end of the discussion to indicate what we are doing to encourage sustainability.  

**It is quite unclear what the focus of this study is**

The focus of the study is to test whether our intervention (training and support embedded within municipal action) leads to increased screening and brief advice activity, compared to no training and no embedding. We have clarified this by adding an hypothesis. 

**It is unclear how the participating cities will be chosen and how the intervention city will be chosen. Including three different “cultures” with only one intervention and one control site for each does not seem to be a robust design.**

Models of international studies across differing cultures have worked well in previous international studies in this area (e.g., WHO studies and the ODHIN study). Our design and statistical measures take into account the multilevel nature of the data. We collect data on potential confounders that can be incorporated in analysis models. To clarify this further, we have added extra text in the discussions. We have added further text describing the selection of cities.

# Peer review: Referee response for version 1

**DOI:** 10.5256/f1000research.12056.r22773

**Published:** 2017-06-23

**Authors:** John B. Saunders

**Affiliations:** 1 Centre for Youth Substance Abuse Research, University of Queensland, Brisbane, QLD, Australia

This paper, which is in the nature of a protocol paper, describes an ambitious three country (six city, 60 primary health care centre) study in Central and South America which seeks to compare the response to the implementation of a comprehensive support strategy for an alcohol screening and brief management approach (and also screening for depression) in comparison with a no support condition, adopting a quasi-experimental design for the comparison. The authors claim that this is the first such controlled study of this type based in middle-income countries. No results of pilot work in these countries are presented but the authors draw upon comparable work undertaken in Europe and the US.    

**Specific Comments**

 The following are points for the authors to consider:

  

1. The introduction starts awkwardly with a description of the alternative ways of conceptualising alcohol misuse. Some of the later statements contradict what is stated earlier about the relationship between alcohol use disorder, alcohol dependence, harmful alcohol use, and risky drinking. What is needed particularly in a scale-up of alcohol screening and intervention is a simple conceptual model, and these exist. There is a natural hierarchy of (1) hazardous/risky drinking (i.e. without harm), (2) harmful drinking (i.e. with harm), and (3) alcohol dependence (where there is a psychobiological driving force to drink not seen in (1) or (2)). Some term the whole spectrum “alcohol misuse” and others “unhealthy alcohol use.” Indeed, such a composite entity was used as the reference standard against which the original AUDIT was gauged. For the definitive version of the paper, I would strongly suggest that the relevant sections of the introduction be reshaped.
2. With regard to the “treatment gap” caution is advised about extrapolating too much from the findings of persons who have treatment to those who have or might not. The latter, although fulfilling the relevant diagnostic criteria, have less severe alcohol misuse and less psychiatric comorbidity and are less complex than those who get into treatment.  
3. Is there any work reported for the three countries selected on the professional supports available to PHC staff? These might include (1) clinical supervision, (2) specialist to generalist support – mentioned in the paper but I can’t see what this would mean - ? on site, by telephone, smartphone apps, (3) peer support – on site or city groupings, (4) administrative support. Is it known what PHC staff would like as an incentive to undertake this work?
4. Reference is made to various scaling-up approaches but the paper would be strengthened by a description and discussion of the theoretical concepts underlying the framework proposed. How does the four-point framework relate to established implementation approaches, which help move organisations into the “early adopter” category? How do the principles of competitive advantage apply, because there will likely be competing proposals for PHC involvement in these countries e.g. childhood immunisation? The use of local “champions” is appropriate but what is the conceptual and empirical basis for this approach? Note that these comments do not refer to the IHI scale-up approach, which is about process.
5. Alcohol screening and intervention outputs will be assessed by the AUDIT-C questionnaires completed and annotated. Depression screening (with the PHQ-9) and intervention is much less emphasised in the body of the paper. Do all the components of the implementation strategy apply equally to this? Will the investigators have access to medical records and PHC throughput data?
6. Overall, this is a very ambitious and complex research program and I became concerned in reading it - in all its complexity - that it might not be achievable with staff who have no prior involvement in research and who are working in resource-constrained health care systems. For example, there is a statement “During all phases of the scale-up, we will document impact on other sectors (education, social care, criminal and justice, etc.) base on resource use measurement.”  This is a huge additional amount of work.
7. The paper would be more convincing (to this reviewer) if it were linked to budgetary inputs. I appreciate that the authors are seeking European Union funding. If this becomes available, the practicalities of doing this study could be set out much more clearly.
8. Although there are approximately 160 references, an impressive number for a protocol paper, there are some surprising omissions, including work which has been specifically addressing implementation of alcohol interventions and including papers that were published from phases of the World Health Organization Implementation Studies and of which the authors of the present paper are also authors.  An example is: Funk M, Wutzke S, Kaner E, Anderson P, Pas L, McCormick R, Gual A, Barfod S, Saunders JB.  A multi-country controlled trial of strategies to promote dissemination and implementation of brief alcohol intervention in primary health care:  Findings of a World Health Organization Collaborative Study. *Journal of Studies on Alcohol* 2005; 66: 379-388. 

I have read this submission. I believe that I have an appropriate level of expertise to confirm that it is of an acceptable scientific standard, however I have significant reservations, as outlined above.

# Response

**Published:** 2017-07-24

**Authors:** Peter Anderson

**Affiliations:** Newcastle University , UK

**The introduction starts awkwardly with a description of the alternative ways of conceptualising alcohol misuse. For the definitive version of the paper, I would strongly suggest that the relevant sections of the introduction be reshaped.**

The nomenclature describing ‘harmful use of alcohol’ and ‘alcohol use disorders’ are cumbersome and somewhat contradictory across organizations and publications, with, for example, three distinct and contradictory concepts of “harmful use” in WHO documents of the last five years alone. As mentioned and referenced in the paper, several of us have argued for, and have published for, clarity, arguing that the term ‘heavy use over time’ can be a simplified replacement descriptor. This ties in well with the epidemiology and simplifies approaches for primary health care clinicians. It fits well with the use of AUDIT-C as the screening instrument, where the overwhelming majority of the variance is due to the consumption items. It is similar to models managing blood pressure. We and the reviewer will probably not come to agreement on this approach. We have carefully re-read the introduction and consider that it accurately reflects the current usages of the terms alcohol use disorder and harmful use of alcohol as used by various institutions and reference bodies. We consider heavy drinking a more appropriate term as the simple conceptual model for use in primary health care, with cut-offs for intervention based on levels at which advice has been shown to be effective (similar to managing blood pressure, as we argue in the section overcoming constraints on PHC activity). The introduction as written reflects the approach and concepts that we are taking in the protocol, and, thus, we do not consider that it is appropriate to adjust.      

**With regard to the “treatment gap” caution is advised about extrapolating too much from the findings**

Yes, this is likely true. Nevertheless, there is an apparent and widespread gap between those who might benefit from advice and treatment and those who get it (like high blood pressure). We have added some words of caution in measuring and reporting treatment gaps. 

**Is there any work reported for the three countries selected on the professional supports available to PHC staff?**

There have been existing studies on testing the impact and implementation of primary health care based screening and advice programmes in Latin American countries, and these are referenced in the paper. We describe the training and support and the city-based adoption and support systems in the paper. We have revised this a little to reflect the reviewer comments. Our collective experience is that many primary health care centres are willing to undertake this work, as they understand the importance of it. Nevertheless, in the project budget, small research fees are allocated for the primary health care centres. If the strategy is proven to work, the expectation is that Departments of Health will adopt the approach as municipal/national strategies.

**Reference is made to various scaling-up approaches but the paper would be strengthened by a description and discussion of the theoretical concepts underlying the framework proposed.**

We have added text on implementation and scale-up literature to the first part of the discussion.

**Will the investigators have access to medical records and PHC throughput data?**

In all three countries, electronic health records are used. Provided ethical and confidentiality agreements are adhered to, anonymous data can be extracted allowing, through confidential identification systems, linkages between questionnaire scores, primary health care consultations and hospitalizations by diagnostic codes. We have added a sentence about this in the discussion.  

**Overall, this is a very ambitious and complex research program and I became concerned in reading it - in all its complexity - that it might not be achievable with staff who have no prior involvement in research and who are working in resource-constrained health care systems.**

We respond by considering that these are false and prejudicial assumptions. The CVs and experiences of the investigators in the three country sites surpass those of many investigators who have implemented similar international studies in ‘high-income’ countries. Further, in the experience of many of the authors of this paper, innovation in implementing screening and brief advice programmes in many Latin American countries surpass that of many high-income countries. Coupled with extensive use of leading technologies, including linked electronic health records, health systems in Latin American countries, whilst relatively resource constrained, are sometimes far ahead than health systems in many ‘high income’ countries.

**There is a statement “During all phases of the scale-up, we will document impact on other sectors (education, social care, criminal and justice, etc.) base on resource use measurement.”  This is a huge additional amount of work.**

This is a misunderstanding. It requires quite minimal data collection from samples of patients. There are then robust tried and tested methodologies to estimate costs per outcome based on the collected data. In any case, these estimates are a requirement within the call for proposals. 

**The paper would be more convincing (to this reviewer) if it were linked to budgetary inputs.**

The purpose of a protocol is to describe the scientific approach. We would not be proposing this study if we did not consider that we had sufficient resources to implement it.

**Although there are approximately 160 references, an impressive number for a protocol paper, there are some surprising omissions**

We have included references to the WHO Phase III and IV studies, which the first author of this paper coordinated whilst he worked with WHO. We have added the Funk *et al.* reference (REF 159).
